# The Association Between Serum Nesfatin-1 Levels and Metabolic Parameters in Women With Polycystic Ovary Syndrome: A Cross-Sectional Study

**DOI:** 10.7759/cureus.106568

**Published:** 2026-04-07

**Authors:** Shubhangi Sharma, Bharti Kawatra, Harpreet K Walia, Monika Jindal

**Affiliations:** 1 Medical Biochemistry, Maharishi Markandeshwar Medical College and Hospital, Kumarhatti-Solan, IND; 2 Biochemistry, Maharishi Markandeshwar Medical College and Hospital, Kumarhatti-Solan, IND; 3 Obstetrics and Gynaecology, Maharishi Markandeshwar Medical College and Hospital, Kumarhatti-Solan, IND

**Keywords:** body mass index, fasting insulin, insulin resistance, metabolic syndrome, nesfatin-1, polycystic ovary syndrome

## Abstract

Background and objective

Polycystic ovary syndrome (PCOS) is a common endocrine disorder associated with metabolic abnormalities such as insulin resistance, obesity, and dyslipidemia. Nesfatin-1, an anorexigenic peptide derived from nucleobindin-2, plays a key role in energy homeostasis and glucose metabolism. However, existing studies report inconsistent findings regarding its levels and metabolic associations in PCOS, and data from small clinical populations remain limited. Hence, this study aimed to evaluate serum nesfatin-1 levels in women with PCOS and examine their association with metabolic and anthropometric parameters, particularly insulin resistance.

Methods

This cross-sectional study included 50 women aged 18-35 years, comprising 25 women with PCOS diagnosed according to the Rotterdam criteria and 25 age-matched healthy controls. Anthropometric parameters were recorded, and fasting blood glucose, fasting insulin, and serum nesfatin-1 levels were measured using standard biochemical methods and enzyme-linked immunosorbent assay (ELISA). Statistical analysis was performed using the independent samples t-test, Pearson correlation, and receiver operating characteristic (ROC) curve analysis.

Results

Women with PCOS exhibited significantly higher fasting blood glucose and fasting insulin levels and significantly lower serum nesfatin-1 levels compared to controls (p < 0.001). Pearson correlation analysis revealed a strong negative correlation between nesfatin-1 and fasting insulin (r = −0.70, p < 0.001) and a moderate negative correlation with fasting blood glucose (r = −0.48, p < 0.001). No significant correlation was observed between nesfatin-1 and BMI (r = −0.17, p = 0.37). ROC curve analysis demonstrated excellent diagnostic performance of nesfatin-1 (area under the curve (AUC) = 0.96).

Conclusions

Serum nesfatin-1 levels are markedly lower in women with PCOS and are negatively correlated with markers of insulin resistance. These findings indicate a possible role for nesfatin-1 in metabolic dysregulation in PCOS and underscore its potential as a biomarker, although additional large-scale and longitudinal studies are needed to confirm these observations.

## Introduction

Polycystic ovarian syndrome (PCOS) is a prevalent endocrine condition among women of reproductive age, impacting around 6-10% of the global female population [1]. Polycystic ovarian morphology, prolonged anovulation, and hyperandrogenism are indicative of the syndrome. PCOS is closely linked to metabolic problems, including obesity, insulin resistance, dyslipidemia, and a higher risk of type 2 diabetes and cardiovascular disease, alongside reproductive complications [2,3]. Insulin resistance is a fundamental component in the pathogenesis of PCOS. In affected women, hyperinsulinemia intensifies reproductive dysfunction and increases ovarian androgen synthesis [4]. Metabolic irregularities linked to PCOS markedly increase the long-term risk of metabolic syndrome and cardiovascular complications [5]. The significance of adipokines and metabolic peptides in the pathophysiology of PCOS has attracted considerable attention in recent years. These peptides modulate insulin sensitivity, glucose metabolism, energy homeostasis, and appetite. The metabolic phenotype in PCOS may be influenced by the dysregulation of multiple metabolic markers [6].

Nesfatin-1, extensively localized in the hypothalamus, pancreas, adipose tissue, and gastrointestinal tract (GIT), plays a significant role in regulating insulin secretion, energy balance, glucose metabolism, and food intake [7,8,9]. Experimental studies have shown that nesfatin-1 affects pancreatic β-cell activity and improves glucose-induced insulin secretion [9]. Despite increasing interest in nesfatin-1 as a metabolic regulator, studies evaluating its circulating levels in PCOS have yielded inconsistent findings, with reports of decreased levels [10], increased levels [11], or unchanged levels [12]. It is also highlighted in recent systematic reviews [10-12]. These inconsistencies may be ascribed to variations in study design, testing methodologies, sample size, and demographic variables.

The present study was designed with three specific objectives: (1) to compare serum nesfatin-1 levels and metabolic parameters between women with PCOS and healthy controls; (2) to evaluate the association between serum nesfatin-1 levels and key metabolic variables, including fasting insulin, fasting blood glucose, and BMI; and (3) to assess the potential diagnostic performance of serum nesfatin-1 in distinguishing women with PCOS from healthy individuals using receiver operating characteristic (ROC) curve analysis.

## Materials and methods

Ethical approval

Ethical approval was secured from the Institutional Ethics Committee of Maharishi Markandeshwar Medical College and Hospital, Kumarhatti, Solan (approval no. MMMCH/IEC/23/782, dated: 27/09/2023). Patients with well-defined PCOS attending the obstetrics and gynecology outpatient department were recruited. The diagnosis was established according to the Rotterdam criteria, factoring in the diagnostic certification provided by the clinical lead.

Study setting and sampling strategy

This was a hospital-based cross-sectional study conducted at the Department of Biochemistry in collaboration with the Department of Obstetrics and Gynaecology at Maharishi Markandeshwar Medical College and Hospital, Kumarhatti-Solan. Participants were recruited from the outpatient department. A convenience sampling method was used, wherein eligible participants who fulfilled the inclusion criteria and provided informed consent were consecutively enrolled during the study period.

Eligibility criteria

Inclusion Criteria

Female patients within the age group of 15-45 years suffering from PCOS as diagnosed by Rotterdam criteria with any of the three features - oligo/anovulation; clinical and biochemical signs of hyperandrogenism; polycystic ovaries on ultrasound defined as ≥12 follicles measuring 2-9 mm in diameter and/or ovarian volume ≥10 cm³ in at least one ovary [5] - were deemed eligible to be included. Only subjects who provided consent and were willing to participate were included in the study.

Exclusion Criteria

Participants with known chronic medical conditions, including diabetes mellitus, hypertension, cardiovascular disease, thyroid disorders, hepatic or renal impairment, and other endocrine disorders, were excluded. Women receiving medications affecting glucose metabolism, hormonal profile, or lipid metabolism (such as insulin, oral hypoglycemic agents, hormonal therapy, or corticosteroids) were also excluded. Pregnant and lactating women were not included in the study.

Healthy controls

Healthy controls comprised age-matched women with regular menstrual cycles from adolescence (menstrual periods lasting up to seven days and cycles ranging from 21 to 35 days), without clinical hyperandrogenism, who were neither medicated nor pregnant, and who provided consent for the study. BMI matching was not performed, and a significant difference in BMI between groups was observed and reported in the results.

Sample size calculation

Sample size was calculated assuming 80% power, a 5% level of significance (α = 0.05), and an expected difference in serum Nesfatin-1 levels between groups based on previously published studies.

The following standard formula was employed:

$n = \frac{Z^2 \times \sigma^2}{d^2}$

where n represents the required sample size, Z is the standard normal deviate (1.96 for 95% confidence interval (CI), σ is the standard deviation (SD) derived from prior studies, and d is the allowable error.

Based on these assumptions, the minimum sample size required was approximately 22 participants per group. To enhance reliability, 25 participants were included in each group. The sample size estimation methodology was based on the standard recommendations established by Charan and Biswas (2013) for biomedical research [13].

Sample collection

A structured questionnaire was used to collect demographic and clinical data from all participants, including age, anthropometric measurements, and clinical history. BMI, waist-hip ratio, and waist-height ratio were calculated using standard methods. Venous blood samples were collected from all participants under fasting conditions on the third day of the menstrual cycle. Samples were obtained under aseptic conditions and processed immediately. Serum was separated by centrifugation and stored at -20 °C for six months until it was analyzed.

Estimation of serum levels of nesfatin-1 and fasting insulin

Serum nesfatin-1 and fasting insulin levels were measured using enzyme-linked immunosorbent assay (ELISA) according to the manufacturer’s protocol (LabReCon ELISA kits, LabRecon®, New Delhi, India). The assays demonstrated high sensitivity and specificity with acceptable intra- and inter-assay precision. ELISA is a well-established immunoassay technique for quantitative detection of peptides and hormones [14]. All laboratory testing was performed at the Biochemistry laboratory of Maharishi Markandeshwar Medical College and Hospital, Kumarhatti-Solan. All instruments, consumables, and equipment were supplied for utilization by the Biochemistry Laboratory of the department.

Assessment of insulin resistance

In the present study, insulin resistance was assessed indirectly using fasting insulin levels. Although indices such as the Homeostatic Model Assessment for Insulin Resistance (HOMA-IR) provide a more standardized measure, they were not calculated in this study.

Statistical analysis

Data were entered into Microsoft Excel and analyzed using SPSS version 20 (IBM Corp., Armonk, NY). Continuous variables were expressed as mean ± SD, and categorical variables as percentages. Between-group comparisons were performed using the independent samples t-test (Welch’s correction applied where appropriate). Pearson's correlation analysis was used to assess relationships between serum nesfatin-1 levels and metabolic parameters. ROC curve analysis was performed to evaluate the diagnostic performance of serum nesfatin-1. The area under the curve (AUC) was used as the primary measure of discrimination. The optimal cut-off value was determined using Youden’s index (sensitivity + specificity − 1). Diagnostic accuracy was interpreted based on AUC, sensitivity, and specificity values. Statistical significance was determined using the independent samples t-test. A p-value < 0.05 was considered statistically significant.

## Results

Anthropometric characteristics

There were no statistically significant differences in age (25.00 ± 3.35 vs. 23.43 ± 2.62 years; p = 0.071) between PCOS cases and controls, indicating appropriate matching between the groups. However, women with PCOS exhibited significantly higher weight (77.47 ± 6.01 vs. 57.20 ± 3.22 kg), BMI (30.06 ± 2.27 vs. 22.25 ± 1.84 kg/m²), waist circumference (37.60 ± 1.71 vs. 29.17 ± 1.34 inches), hip circumference (42.73 ± 1.95 vs. 38.47 ± 1.20 inches), waist-hip ratio (0.88 ± 0.06 vs. 0.76 ± 0.04), and waist-height ratio (0.60 ± 0.03 vs. 0.46 ± 0.02) compared to controls (all p < 0.001). Compared with controls, women with PCOS had significantly higher BMI (Δ = 7.81 kg/m²; 95% CI: 6.64-8.98), waist circumference (Δ = 8.43 inches; 95% CI: 7.56-9.30), and waist-height ratio (Δ = 0.14; 95% CI: 0.13-0.15), indicating greater central adiposity (Table 1).

**Table 1:** Anthropometric characteristics of PCOS patients and controls Group comparisons were performed using the independent samples t-test. A p-value < 0.05 was considered statistically significant PCOS: polycystic ovarian syndrome; SD: standard deviation; BMI: Body mass index

Characteristics	PCOS cases (n = 25), mean ± SD	Controls (n = 25), mean ± SD	t-value	P-value
Age (years)	25.00 ± 3.35	23.43 ± 2.62	1.85	0.071
Weight (kg)	77.47 ± 6.01	57.20 ± 3.22	14.86	< 0.001
BMI (kg/m²)	30.06 ± 2.27	22.25 ± 1.84	13.36	< 0.001
Waist circumference (inches)	37.60 ± 1.71	29.17 ± 1.34	19.4	< 0.001
Hip circumference (inches)	42.73 ± 1.95	38.47 ± 1.20	9.3	< 0.001
Waist–hip ratio	0.88 ± 0.06	0.76 ± 0.04	8.32	< 0.001
Waist–height ratio	0.60 ± 0.03	0.46 ± 0.02	19.41	< 0.001

Biochemical parameters

Women with PCOS exhibited significant alterations in metabolic parameters compared to controls. Fasting blood glucose levels were significantly higher in the PCOS group (96.77 ± 3.42 vs. 86.03 ± 1.88 mg/dL; p < 0.001). Similarly, fasting insulin levels were markedly elevated in PCOS patients (2.40 ± 0.43 vs. 0.73 ± 0.22 ng/mL; p < 0.001), indicating the presence of insulin resistance. In contrast, serum nesfatin-1 levels were significantly reduced in women with PCOS compared to controls (748.33 ± 77.39 vs. 895.50 ± 47.51 pg/mL; p < 0.001). These findings demonstrate a pattern of metabolic dysregulation in PCOS, characterized by hyperglycemia, hyperinsulinemia, and decreased nesfatin-1 concentrations. Fasting insulin levels were markedly higher in women with PCOS compared to controls (Δ = 1.67 ng/mL; 95% CI: 1.48-1.86), consistent with insulin resistance, although standardized indices such as HOMA-IR were not assessed (Table 2).

**Table 2:** Biochemical and metabolic parameters in women with PCOS and controls Group comparisons were performed using the independent samples t-test. A p-value < 0.05 was considered statistically significant PCOS: polycystic ovarian syndrome; SD: standard deviation

Parameters	PCOS cases (n = 25), mean ± SD	Controls (n = 25), mean ± SD	t-value	P-value	Degree of freedom
Fasting blood glucose	96.77 ± 3.42	86.03 ± 1.88	13.76	< 0.001	37
Fasting insulin (ng/ml)	2.40 ± 0.43	0.73 ± 0.22	17.29	< 0.001	36
Nesfatin-1 (pg/ml)	748.33 ± 77.39	895.50 ± 47.51	-8.1	< 0.001	40

Correlational analysis

Pearson's correlation analysis revealed a strong negative correlation between serum nesfatin-1 levels and fasting insulin (r = −0.70, p < 0.001), indicating that higher insulin levels are associated with lower nesfatin-1 concentrations. A moderate negative correlation was observed between nesfatin-1 and fasting blood glucose (r = −0.48, p < 0.001). Additionally, fasting insulin showed a moderate positive correlation with fasting blood glucose (r = 0.49, p < 0.001), suggesting a parallel increase in these metabolic parameters (Table 3).

**Table 3:** Correlation between nesfatin-1 and fasting insulin, nesfatin-1 and fasting blood glucose, and fasting insulin and fasting blood glucose Correlation analysis was performed using Pearson’s correlation coefficient. A p-value < 0.05 was considered statistically significant

Variables	r value	P-value	Interpretation
Nesfatin-1 vs. fasting insulin	−0.70	< 0.001	Strong negative (highly significant)
Nesfatin-1 vs. fasting blood glucose	−0.48	< 0.001	Moderate negative (significant)
Fasting insulin vs. fasting blood glucose	0.49	< 0.001	Moderate positive (significant)

Association of nesfatin-1 with BMI

No significant association was observed between serum nesfatin-1 levels and BMI (r = −0.17; 95% CI: −0.53 to 0.24), indicating a weak and non-significant relationship between nesfatin-1 and adiposity in the study population (Table 4).

**Table 4:** Correlation analysis of serum nesfatin-1 with BMI Correlation analysis was performed using Pearson’s correlation coefficient. A p-value < 0.05 was considered statistically significant BMI: body mass index

Parameter	Correlation coefficient (r)	P-value
BMI	−0.17	0.37

Comparison of serum nesfatin-1 levels

Women with PCOS demonstrated significantly lower nesfatin-1 levels compared to controls (748.33 ± 77.39 vs. 895.50 ± 47.51 pg/mL; p < 0.001).

Correlation between nesfatin-1 and fasting insulin

**Figure 1:**
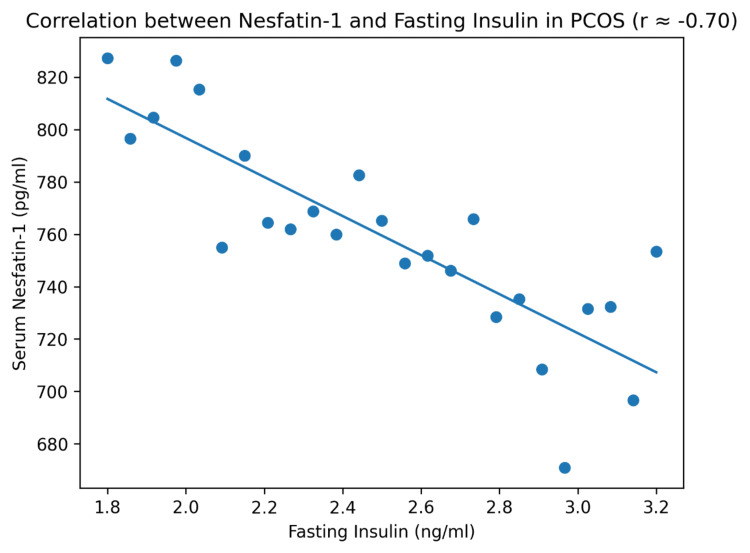
Correlation between nesfatin-1 and fasting insulin in PCOS Correlation was assessed using Pearson’s correlation coefficient. The line represents the linear regression fit. A p-value < 0.05 was considered statistically significant PCOS: polycystic ovarian syndrome

The relationship between serum nesfatin-1 and fasting insulin levels in women with PCOS is illustrated in Figure 1. The scatter plot demonstrates a strong negative correlation between these variables (r = −0.70, p < 0.001), supporting the findings of the correlation analysis.

Diagnostic performance of nesfatin-1

**Figure 2:**
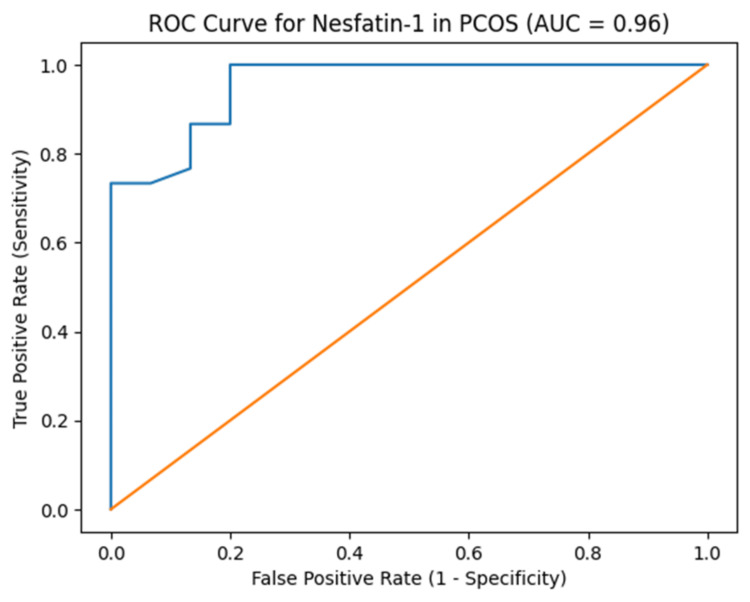
ROC curve illustrating serum nesfatin-1's diagnostic performance for PCOS AUC = 0.95 (95% CI: 0.90-0.99). Optimal cut-off = 799 pg/mL, with sensitivity = 100% and specificity = 80% Receiver operating characteristic; PCOS: polycystic ovarian syndrome; AUC: area under the curve; CI: confidence interval

The diagnostic performance of nesfatin-1 is depicted in Figure 2. ROC curve analysis demonstrated good diagnostic performance of serum nesfatin-1 in distinguishing women with PCOS from healthy controls, with an AUC of 0.95 (95% CI: 0.90-0.99). The optimal cut-off value, determined using Youden’s index, was 799 pg/mL, yielding a sensitivity of 100% and specificity of 80%.

## Discussion

The current study observed considerably lower serum nesfatin-1 levels in women with PCOS compared to healthy controls, alongside notable changes in metabolic parameters, including increased fasting blood glucose, fasting insulin, and indicators of central obesity, as shown in Table 2. The reduced nesfatin-1 levels observed in this study may reflect altered metabolic signaling and could be associated with insulin resistance through effects on pancreatic β-cell function and glucose homeostasis. However, these mechanisms cannot be confirmed within the scope of the present cross-sectional study. This study demonstrated significantly reduced circulating nesfatin-1 levels in patients with PCOS. Nesfatin-1, originating from nucleobindin-2 (NUCB2), is identified as an anorexigenic peptide that plays a role in appetite regulation, glucose homeostasis, and insulin secretion. Prior research has shown that nesfatin-1 augments glucose-stimulated insulin production and regulates pancreatic β-cell activity, therefore facilitating metabolic equilibrium [3,10]. The reduced levels found in this study may consequently indicate compromised metabolic signaling in PCOS.

The observed reduction in nesfatin-1 levels aligns with prior studies by Deniz et al. [7] and Alp et al. [8], which also reported significantly reduced circulating nesfatin-1 concentrations in women with PCOS. These investigations indicated that diminished nesfatin-1 levels may lead to insulin resistance and metabolic abnormalities. Nevertheless, certain investigations have indicated contradictory findings, revealing either elevated or stable nesfatin-1 levels in populations with PCOS [11]. The conflicting findings across studies may be attributed to heterogeneity in study populations, differences in BMI distribution, variability in insulin resistance profiles, assay-related differences in nesfatin-1 measurement, and variations in sample size.

The current investigation revealed that women with PCOS demonstrated markedly elevated fasting insulin levels, signifying insulin resistance, a fundamental characteristic in the pathophysiology of PCOS. Hyperinsulinemia is recognized to exacerbate hyperandrogenism and facilitate metabolic dysfunction [4]. The Pearson correlation analysis indicated a significant negative correlation between nesfatin-1 and fasting insulin (r = −0.70, p < 0.001), as shown in Table 3, suggesting that lower nesfatin-1 levels may be associated with increased insulin resistance. This finding is consistent with previous experimental and clinical studies suggesting that nesfatin-1 is involved in insulin signaling and glucose metabolism [3].

A moderate negative correlation was observed between nesfatin-1 and fasting blood glucose (r = −0.48, p < 0.001), as shown in Table 3, suggesting a possible link between reduced nesfatin-1 levels and impaired glucose homeostasis. The findings indicate that nesfatin-1 may play a role in the initial disruptions of glucose metabolism in PCOS. Anthropometric assessment, as shown in Table 1, indicated markedly elevated BMI and central obesity metrics in women with PCOS. No significant association was observed between serum nesfatin-1 levels and BMI (r = −0.17; 95% CI: −0.53 to 0.24), indicating a weak and non-significant relationship between nesfatin-1 and adiposity in the study population. This indicates that although obesity is significantly linked to PCOS, the connection between nesfatin-1 and adiposity may be complex and affected by other metabolic variables. Prior research has indicated inconsistent correlations between nesfatin-1 and BMI [5,9].

Our findings revealed that serum nesfatin-1 had good diagnostic performance in differentiating PCOS patients from controls, evidenced by an AUC of 0.96, as exhibited in Figure 2. This signifies a substantial level of sensitivity and specificity, implying that nesfatin-1 could function as a potential biomarker for PCOS. Previous investigations have revealed analogous findings, underscoring the clinical significance of metabolic peptides in the diagnosis of PCOS [8]. The strong inverse correlation between nesfatin-1 and insulin suggests a possible role of nesfatin-1 in modulating insulin sensitivity in PCOS. The findings of this study indicate that low levels of nesfatin-1 correlate with insulin resistance, disrupted glucose metabolism, and central obesity in PCOS. The inverse correlation between nesfatin-1 and insulin levels may be associated with metabolic alterations in PCOS and warrants further investigation.

The findings of the present study should be interpreted with caution in light of certain limitations. The relatively small sample size may limit the generalizability of the results and increase the possibility of sampling variability. In addition, the cross-sectional design precludes causal inference between nesfatin-1 levels and metabolic alterations in PCOS. The absence of a standardized index of insulin resistance, such as HOMA-IR, further limits mechanistic interpretation, as insulin resistance was assessed using fasting insulin levels alone. Furthermore, the significant difference in adiposity between the PCOS and control groups may have influenced the observed metabolic parameters, including nesfatin-1 levels, and should be considered while interpreting these associations.

Strengths

This study assessed nesfatin-1 levels in conjunction with detailed anthropometric and metabolic characteristics, offering a thorough evaluation of metabolic dysfunction in PCOS. Furthermore, correlation and ROC analyses enhanced the clinical significance of the results.

Limitations

The study is constrained by its modest sample size and cross-sectional methodology, which preclude causal conclusions. A key limitation of the study is the absence of HOMA-IR calculation for the assessment of insulin resistance. Although fasting glucose and insulin were measured, the use of insulin values in ng/mL limited the direct calculation of HOMA-IR, which requires insulin in µIU/mL. Future studies incorporating standardized insulin measurements would allow a more precise assessment of insulin resistance. Subsequent research utilizing larger sample sizes and longitudinal methodologies is necessary to corroborate these findings.

## Conclusions

The present study demonstrates that serum nesfatin-1 levels are significantly reduced in women with PCOS and are associated with key metabolic parameters, including fasting insulin and glucose levels. These findings suggest a possible link between nesfatin-1 and metabolic dysregulation in PCOS. However, given the cross-sectional design, small sample size, and differences in adiposity between groups, the results should be interpreted with caution. Further large-scale and longitudinal studies are required to better understand the role of nesfatin-1 in PCOS.
